# Trends and Disparities in Initiation of Buprenorphine in US Emergency Departments, 2013-2022

**DOI:** 10.1001/jamanetworkopen.2024.35603

**Published:** 2024-09-26

**Authors:** Neeraj Chhabra, Dale Smith, Grayson Dickinson, Lily Caglianone, R. Andrew Taylor, Gail D’Onofrio, Niranjan S. Karnik

**Affiliations:** 1 Department of Emergency Medicine, University of Illinois Chicago; 2 AI.Health4All Center for Healthy Equity using Machine Learning and Artificial Intelligence, College of Medicine, University of Illinois Chicago; 3 Institute for Research on Addictions, University of Illinois Chicago; 4 College of Osteopathic Medicine, William Carey University, Hattiesburg, Mississippi; 5 Department of Psychiatry, University of Illinois Chicago; 6 Department of Emergency Medicine, Yale University School of Medicine, New Haven, Connecticut; 7 Department of Medicine, Yale School of Medicine, New Haven, Connecticut; 8 Department of Epidemiology (Chronic Disease), Yale School of Public Health, New Haven, Connecticut

## Abstract

This cross-sectional study assesses trends in buprenorphine initiation in US emergency departments and demographic and socioeconomic factors associated with its initiation.

## Introduction

Increasing the availability of buprenorphine is an important component of the public health response to the opioid epidemic. As a low-barrier point of contact, emergency departments (EDs) represent a critical setting for buprenorphine initiation, although practices are varied, with local disparities in buprenorphine initiation.^1,2,3^

The evolution in ED-initiated buprenorphine and existence of disparities in its application remain undetermined at a national level. We evaluated national trends in ED buprenorphine initiation and demographic and socioeconomic factors associated with its initiation.

## Methods

This retrospective cross-sectional study used encounter-level ED buprenorphine administration and prescribing data from January 1, 2013, to December 31, 2022, from Epic Cosmos, a nationally representative research system encompassing over 1100 hospitals and 7.2 billion encounters. We queried Cosmos for ED encounters involving administration or prescribing of any buprenorphine formulation. To account for year-to-year variation, descriptive results were standardized to a denominator of ED encounters containing opioid-related *ICD-10* billing codes (eAppendix in Supplement 1). The University of Illinois Chicago institutional review board exempted the study, with a waiver of informed consent, because it was non–human participant research. The study followed the STROBE reporting guideline.

To examine disparities, we fit a multivariate binary logistic regression model using sex, ethnicity, race, age, urban or rural designation, insurance, and social vulnerability index to an outcome of buprenorphine administration or prescribing over the most recent 3 years. Python, version 3.12.0 was used for analysis. Two-sided *P* < .05 was significant.

## Results

Of 1 632 674 opioid-related encounters from 2013 to 2022, 217 832 involved initiation of buprenorphine (mean [SD] age, 49.0 [15.3] years; 44.3% female and 55.7% male; 2.0% American Indian or Alaska Native, 0.6% Asian, 18.5% Black, 0.3% Native Hawaiian or Other Pacific Islander, 74.9% White, and 3.6% other; 6.8% Hispanic, 89.0% non-Hispanic, and 4.1% unspecified). Emergency encounters involving buprenorphine increased from 2.75% of opioid-related encounters in 2013 to 27.3% in 2022, with increases in administration and prescribing (Figure). Of 745 289 opioid-related ED encounters across 452 684 unique patients in the most recent 3 years, 16.1% were administered (14.8%) or prescribed (5.7%) buprenorphine.

**Figure.**
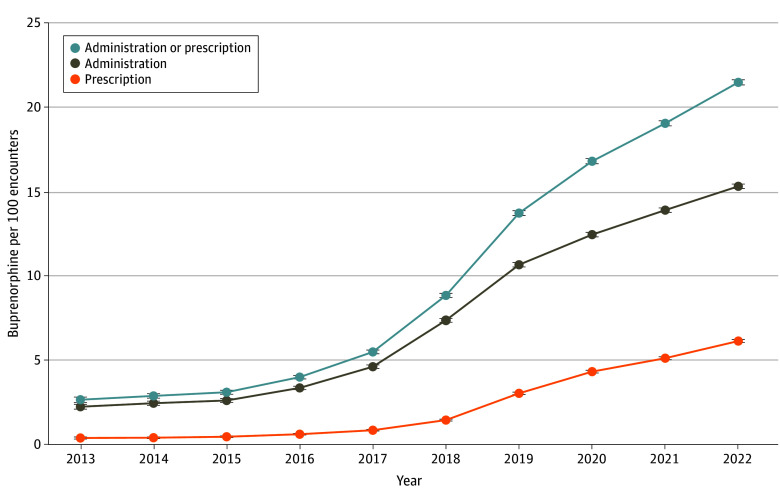
Emergency Department Encounters Involving Buprenorphine Administration and/or Prescription

Adjusted analyses (Table) indicated elevated odds for receiving buprenorphine among males (odds ratio [OR], 1.17; 95% CI, 1.15-1.18). White participants had increased odds of receiving buprenorphine compared with participants identified as American Indian or Alaska Native (OR, 0.91; 95% CI, 0.87-0.94), Asian (OR, 0.83; 95% CI, 0.77-0.90), Black or African American (OR, 0.71; 95% CI, 0.70-0.72), Native Hawaiian or Other Pacific Islander (OR, 0.72; 95% CI, 0.64-0.82), or other race (OR, 0.86; 95% CI, 0.83-0.90).

**Table.** Crude and Adjusted Odds Ratios for Administration of Buprenorphine

Predictor	Odds ratio (95% CI)	
	Adjusted^a^	Crude
Sex		
Female	1 [Reference]	1 [Reference]
Male	1.17 (1.15-1.18)	1.16 (1.15-1.18)
Ethnicity^b^		
Hispanic	1 [Reference]	1 [Reference]
Non-Hispanic	1.03 (1.00-1.05)	0.94 (0.92-0.96)
RUCA^c^		
Rural	1 [Reference]	1 [Reference]
Urban	0.98 (0.96-1.00)	0.96 (0.94-0.99)
Age, by 1-y increase	0.99 (0.99-0.99)	0.99 (0.98-0.99)
Race^b^		
American Indian or Alaska Native	0.91 (0.87-0.94)	0.93 (0.89-0.97)
Asian	0.83 (0.77-0.90)	0.97 (0.90-1.04)
Black or African American	0.71 (0.70-0.72)	0.70 (0.69-0.71)
Native Hawaiian or Other Pacific Islander	0.72 (0.64-0.82)	0.77 (0.68-0.87)
White	1 [Reference]	1 [Reference]
Other^d^	0.86 (0.83-0.90)	0.92 (0.89-0.96)
Social Vulnerability Index, quartile^e^		
1		
2	0.97 (0.96-0.99)	0.96 (0.95-0.98)
3	0.94 (0.92-0.95)	0.93 (0.90-0.94)
4	1.00 (0.98-1.01)	0.92 (0.91-0.94)
Insurance		
Medicaid	1 [Reference]	1 [Reference]
Medicare	0.78 (0.76-0.80)	0.61 (0.60-0.63)
Private^f^	0.93 (0.91-0.94)	0.84 (0.83-0.85)
Self-pay	0.59 (0.57-0.61)	0.63 (0.61-0.65)

Abbreviation: RUCA, rural-urban commuting area code.

^a^ Adjusted for age, sex, race, ethnicity, RUCA, social vulnerability index, and insurance. All adjusted *P* values were <.001 except RUCA (*P* = .049) and the social vulnerability index fourth quartile (*P* = .02).

^b^ Race and ethnicity were ascertained from Epic Cosmos.

^c^ RUCA designation was dichotomized, with rural areas including a RUCA of 8 to 10 as defined by Epic Cosmos.

^d^ Other was defined by Epic Cosmos and was not broken down further.

^e^ A social vulnerability index of 1 indicates the least social vulnerability and 4, the most social vulnerability.

^f^ Includes third-party liability, Tricare, US Family Health Plan, US Department of Veterans Affairs, and worker’s compensation as defined by Epic Cosmos.

## Discussion

This study showed that while buprenorphine administration and prescribing in the ED increased over the past decade, its use was uncommon in patients with opioid-related encounters and disparities existed in its use. Buprenorphine was prescribed in only 5.7% of opioid-related ED encounters from the most recent 3 years, and more patients received a dose of buprenorphine than a prescription. One-time administration without maintenance prescription may negatively impact treatment retention, as withdrawal symptoms are likely to be delayed with 1-time dosing. A 7-day extended-release injectable formulation can be considered when barriers to prescribing exist.^4^

Despite efforts to expand buprenorphine use, broad adoption of low-threshold buprenorphine in EDs is limited by lack of local follow-up, practitioner hesitancy, stigma surrounding substance use treatment, and insufficient resources for addressing co-occurring social determinants of health.^5,6^ Addressing barriers and reducing disparities in buprenorphine use in EDs is imperative to harness its proven efficacy in decreasing opioid use and preventing premature death. Study limitations are the retrospective design, lack of availability for certain variables, and use of billing *ICD-10* codes in case identification, which may lack sensitivity.

This study found racial disparities in buprenorphine initiation and that female sex was associated with decreased initiation of buprenorphine. Further research and interventions are warranted to elucidate and mitigate the factors contributing to these disparities, thereby enhancing the ED’s capacity as an intervention point in the opioid epidemic.

## Notes

Supplement 1. **eAppendix. **Opioid-related *ICD-10* codes

Supplement 2. Data Sharing Statement
