# Supplementary material for: Trends and Disparities in Initiation of Buprenorphine in US Emergency Departments, 2013-2022
Source: JAMA Netw Open. 2024 Sep 26;7(9):e2435603. doi: 10.1001/jamanetworkopen.2024.35603 (PMC11428009; doi:10.1001/jamanetworkopen.2024.35603)
Supplement: Supplement 1. — eAppendix. Opioid-related ICD-10 codes [file jamanetwopen-e2435603-s001.pdf]

Supplemental Online Content

Chhabra N, Smith D, Dickinson G, et al. Trends and disparities in initiation of buprenorphine in US emergency departments, 2013-2022. *JAMA Netw Open.* 2024;7(9):e2435603. doi:10.1001/jamanetworkopen.2024.35603

eAppendix. Opioid-related *ICD-10* codes

This supplemental material has been provided by the authors to give readers additional information about their work.

© 2024 Chhabra N et al. *JAMA Network Open.*

Appendix. Opioid-related ICD-10 codes

Abuse or dependence F11 series: Opioid-related disorders (except F11.21)

Poisoning, including self-harm T40.0X1, 0X2, 0X3, 0X4: Poisoning by opium-accidental, intentional self-harm, assault, or undetermined

T40.1X1, 1X2, 1X3, 1X4: Poisoning by heroin-accidental, intentional self-harm, assault, or undetermined

T40.2X1, 2X2, 2X3, 2X4: Poisoning by other opioids-accidental, intentional self-harm, assault, or undetermined

T40.3X1, 3X2, 3X3, 3X4: Poisoning by methadone-accidental, intentional self-harm, assault, or undetermined

T40.4X1, 4X2, 4X3, 4X4: Poisoning by other synthetic narcotics-accidental, intentional self-harm, assault, or undetermined

T40.601-T40.604: Poisoning by unspecified narcotics-accidental, intentional self-harm, assault, or undetermined

T40.691-T40.694: Poisoning by other narcotics-accidental, intentional self-harm, assault, or undetermined

Abbreviation: ICD-10-CM, International Classification of Diseases, Tenth Revision, Clinical Modification

Derived from Weiss AJ (IBM Watson Health), McDermott KW (IBM Watson Health), Heslin KC (AHRQ). Opioid-Related Hospital Stays Among Women, 2016. HCUP Statistical Brief #247. January 2019. Agency for Healthcare Research and Quality, Rockville, MD. www.hcup-us.ahrq.gov/reports/statbriefs/sb247-Opioid-Hospital-Stays-Women.pdf.

© 2024 Chhabra N et al. *JAMA Network Open.*
