# Supplementary material for: Trends and Disparities in Initiation of Buprenorphine in US Emergency Departments, 2013-2022
Source: JAMA Netw Open. 2024 Sep 26;7(9):e2435603. doi: 10.1001/jamanetworkopen.2024.35603 (PMC11428009; doi:10.1001/jamanetworkopen.2024.35603)
Supplement: Supplement 2. — Data Sharing Statement [file jamanetwopen-e2435603-s002.pdf]

Data Sharing Statement

Chhabra. Trends and Disparities in Initiation of Buprenorphine in US Emergency Departments, 2013-2022. *JAMA Netw Open*. Published September 26, 2024.
doi:10.1001/jamanetworkopen.2024.35603

Data
Data available: No

Additional Information
Explanation for why data not available: Data is maintained and manage by Epic Systems Corporation (Verona, WI, USA). Requests for data should be directed to Epic Systems.
